# Associations of the gut microbiome and inflammatory markers with mental health symptoms: a cross-sectional study on Danish adolescents

**DOI:** 10.1038/s41598-025-94687-7

**Published:** 2025-03-26

**Authors:** Aisha Alayna Brown, Michael Widdowson, Sarah Brandt, Parisa Mohammadzadeh, Julie B. Rosenberg, Jens Richardt Møllegaard Jepsen, Bjørn H. Ebdrup, María Hernández-Lorca, Klaus Bønnelykke, Bo Chawes, Jakob Stokholm, Jonathan Thorsen, Parvaneh Ibrahimi, Xuanji Li, Søren Johannes Sørensen, Morten Arendt Rasmussen

**Affiliations:** 1 Section of Global Health, Department of Public Health, University of Copenhagen, Copenhagen, Denmark; 2 COPSAC, Copenhagen Prospective Studies on Asthma in Childhood, Herlev and Gentofte Hospital, University of Copenhagen, Copenhagen, Denmark; 3 Department of Food Science, University of Copenhagen, Copenhagen, Denmark; 4 Department of Clinical Medicine, Faculty of Health and Medical Sciences, University of Copenhagen, Copenhagen, Denmark; 5 Center for Neuropsychiatric Schizophrenia Research (CNSR), Mental Health Centre Glostrup, University of Copenhagen, Glostrup, Denmark; 6 Child and Adolescent Mental Health Center, Copenhagen University Hospital–Mental Health Services CPH, Copenhagen, Denmark; 7 Department of Pediatrics, Slagelse Hospital, Slagelse, Denmark; 8 Section of Microbiology, School of Life Sciences, University of Zhejiang, Hangzhou, China; 9 Section of Microbiology, Department of Biology, University of Copenhagen, Copenhagen, Denmark; 10 Department of Health Technology, Technical University of Denmark, Lyngby, Denmark

**Keywords:** Gut microbiome, GlycA, ADHD, Mental health, Neurodevelopmental disorders, Inflammatory biomarkers, Neurological manifestations, Chronic inflammation, Bacteria, Biomarkers, ADHD, Anxiety, Depression

## Abstract

Attention-deficit/hyperactivity disorder (ADHD) is a common neurodevelopmental disorder that often persists into adulthood and is accompanied by comorbid mental health problems. This cross-sectional cohort study analyzed 411 18-year-olds from the Danish COPSAC_2000_ birth cohort to investigate the relationship between the gut microbiome, fasting and postprandial systemic inflammation, ADHD symptoms, and symptoms of anxiety, stress, and depression. ADHD was assessed using the Adult ADHD Self-Report Scale (ASRS), while depression, stress, and anxiety were evaluated with the Depression, Anxiety, and Stress Scale 21 (DASS-21). Fecal metagenomic data and inflammation levels, measured as glycosylated protein A (GlycA), were analyzed following a standardized meal challenge. In males, higher ADHD symptom scores correlated significantly with increased abundance of a tryptophan biosynthesis pathway (MetaCyc Metabolic Pathways Database) and elevated fasting and postprandial GlycA levels (*p* < 0.05). While the severity of depression, anxiety, and stress symptoms showed weak associations with GlycA and the gut microbiome, our findings indicate a significant link between ADHD symptoms and postprandial inflammation, warranting further investigation into underlying mechanisms.

**Supplementary Information:**

The online version contains supplementary material available at 10.1038/s41598-025-94687-7.

## Introduction

Globally, 2.5% of adults and 5.9% of children and adolescents have attention-deficit/hyperactivity disorder (ADHD)^1^. ADHD is a neurodevelopmental disorder characterized by three core symptoms: inattention, hyperactivity, and impulsivity. It is often accompanied by impairments in level of functioning, academic performance, social interactions, and other everyday activities^2^.

Brain-gut microbiome interactions are one mechanism that has been implicated in ADHD^3–7^. The gut microbiome consists of the community of microbes (the microbiota), their collective genes (the metagenome), gene products, and associated metabolites in their defined environment^8^. They form a complex ecosystem and are dependent on environmental factors as well as competition with each other^9^. Disruption or imbalance of the gut microbial community, often referred to as dysbiosis, can be detrimental to health^10^.

Along with the gut microbiome, ADHD has been linked to inflammatory cytokines and polymorphisms in inflammation-related genes^11^. ADHD, and many of its comorbidities, including anxiety and depression, have also been correlated with inflammatory disorders (e.g., psoriasis, asthma, allergic rhinitis)^12,13^. Increased stress, which is associated with ADHD, has been linked to increased inflammation as well^13^. Although findings point towards inflammation as a contributor to the neurodevelopment of ADHD^11^, low-grade inflammation has been shown in ADHD adults^14^. Pro-inflammatory gut microbes may be contributing to elevated inflammation levels characteristic of people with ADHD^15^. However, the particular role of gut microbiota remains unknown^16,17^.

Depression and anxiety are increasingly prevalent worldwide, contributing to significant individual and societal burdens. Addressing subclinical levels of anxiety and depression is an important step in reducing the prevalence of the disorders through preventative measures^18^. This study aimed to do this by uncovering potential links between subclinical neuropsychiatric symptoms, inflammation, and the gut microbiome. Furthermore, we aimed to investigate how the composition of the gut microbiome is associated with ADHD symptoms, as measured by the ASRS, and symptoms of stress, anxiety, and depression, as measured by the DASS-21 in the COPSAC_2000_ birth cohort. In this cohort, all participants have a mother who was diagnosed with asthma. Additionally, we examined the role of fasting and postprandial inflammation in these associations within the COPSAC_2000_ birth cohort. We investigated inflammation levels during a post-prandial nutritional stress test, using a mixture of carbohydrates, lipids, and protein to induce an inflammatory response to quantify the body’s ability to cope with an acute, stressful environment. Given sex differences for both the etiology of ADHD^19^, the gut microbiome composition^20^ and adiposity and hence inflammation^21,22^, our analyses were stratified by gender.

## Materials and methods

### COPSAC_2000_ birth cohort

COPSAC_2000_ is a single-center prospective birth cohort with 411 participants followed from birth^23^. All participants in the cohort have a mother who has a history of doctor-diagnosed asthma and is, therefore, not totally representative of the Danish population. Exclusion criteria included severe congenital anomaly, a gestational age of less than 36 weeks, a need for mechanical ventilation, or a lower respiratory tract infection at birth. The cohort was followed from the age of 4 weeks onwards with semi-annual visits to the research clinic until 7 years, and again at 13 and 18 years. All data used in this study originates from the 18-year visit. As this study utilizes a previously established cohort in which data collection had already occurred, we were inherently limited in the availability of select measures. However, we carefully chose from the available measures to best address our research objectives. The 18-year visit included self-report questionnaires examining mental health status, a nutritional stress test, and microbiome sampling of the participants, all of which are used in this study. It is important to note that this study did not utilize formal ADHD diagnoses and none of the participants were grouped based on a clinical ADHD diagnosis. All participants were provided informed consent during the study.

### Questionnaires

#### Adult ADHD Self-Report scale (ASRS)

The participants completed an electronic version of the ASRS symptoms checklist. The ASRS contains 18 questions corresponding to 18 symptoms consistently found in adults with ADHD according to the American Psychiatric Association’s Diagnostic and Statistical Manual of Mental Disorders (DSM-IV-TR)^24^. There are 2 parts to the questionnaire. Part A contains 6 questions with a total score ranging from 0 to 6 and is the main section used to determine if the participant had symptoms consistent with ADHD or not. Part B consists of 12 questions with a total score ranging from 0 to 12, providing information on the participants’ symptoms severity and specific subtypes. The questionnaire is scored on a 5-point scale with never, rarely, sometimes, often, and very often, based on how frequently they experienced the symptoms over the last 6 months. Questions 1–3, 9, 12, 16, and 18 were scored 1 for sometimes, often, or very often, and 0 for never or rarely. The remaining questions were scored 1 for often or very often, and 0 for sometimes, rarely, and never. Participants were included in the ASRS + group if they scored higher than a 3 on Part A of the ASRS, which is shown to be an accurate predictor of ADHD in adults when used as a screening tool^24–26^. Participants that scored a 3 or lower on Part A of the ASRS are assigned to the ASRS- group. The ASRS questionnaire can be separated into inattentive (questions 1, 2, 3, 4, 7, 8, 9, 10, 11), motor hyperactive/impulsivity (questions 5, 6, 12, 13, 14), and verbal hyperactivity/impulsivity (questions 15, 16, 17, 18) subscales as described by Stanton et al. [2018]. In this study, these three subscales will be referred to as ADHD symptom presentations as described by Ryan and Sadek [2023]. The ASRS has been validated as a screening tool for ADHD in Scandinavia^29^, and it has been successfully used in a large population-based study in Denmark^30^.

#### Depression, anxiety, and stress scale 21 (DASS-21)

The participants completed the DASS-21 to assess depression, anxiety, and stress symptom levels. This scale is composed of 21 questions, 7 pertaining to each category (depression, anxiety, and stress)^31^. This scale was not intended to diagnose any of these conditions, rather it outlined anxiety, depression, and stress symptom severity^32^. Therefore, subscale scores of each category (depression, anxiety, and stress) are used for downstream analysis. The depression and anxiety portions of this scale have been demonstrated to be consistent with established screening tools for anxiety and depression such as the Generalized Anxiety Disorder-7 scale, Center for Epidemiological Studies-Depression Scale, state trait anxiety inventory, self-rating depression scale, the Beck Depression Inventory, the Beck Anxiety Inventory^33–35^.

### Biological sampling

#### Blood sampling and nutritional stress test

The first blood sample was extracted following a minimum of an 8-hour fasting period. After the first sample was collected, the participants received a standardized meal of macronutrients (60 g palm olein, 75 g glucose, and 20 g dairy protein in a total volume of 400 mL). This intake has been proposed as nutritional “stress testing”^36^. The meal was consumed in 30 minutes or less. Once half was consumed, the timer for the blood draws was set. The post-prandial fluctuations of these macronutrients were evaluated through blood sampling at 15, 30, 60, 90, 120, 180, and 240 minutes after intake. In this study, we only used measurements up to 2 hours, as that was when the levels peaked. Fasting and post-nutritional stress testing levels of GlycA were collected to evaluate the degree of baseline and inducible systemic inflammation. GlycA was measured using a targeted high-throughput NMR metabolomics platform (Nightingale Health Ltd., Helsinki, Finland) as described by Ebrahimi et al. (2024). GlycA levels were measured from the plasma using Proton Nuclear Magnetic Resonance (^1^H-NMR) to scan the glycan region of acute phase proteins such as α1-acid glycoprotein, haptoglobin, α1-antitrypsin and α1-antichymotrypsin^38^.

GlycA is a biomarker for acute and systemic inflammation. Although a novel biomarker, GlycA has previously been associated with established markers for inflammation such as tumor necrosis factor-α, fibrinogen, CRP and IL-6^38^. Current evidence suggests that GlycA may be useful in the same applications as hs-CRP. Although more research is needed, it is suggested that GlycA may be a more accurate measure of the inflammatory state since it integrates the measure of multiple acute phase proteins instead of just one, reducing the intra-individual variability^38,39^.

Research on GlycA and mental health are severely lacking, however other comparable inflammatory markers are known to play a central role in mental health symptoms and the risk of developing neurodevelopmental disorders^13,40–42^.

### Microbiome analysis

#### Feces samples

Up to 20 mL of feces were collected for analysis of the fecal microbiota at the 18 year-visit. The fecal samples were collected by the participants either at the research clinic or in their own homes using detailed instructions. The samples were then sent directly to the Department of Biology, University of Copenhagen. Here, they were stored at – 80 °C for later microbiome analysis. Genomic DNA of 200–250 mg fecal samples was extracted with the NucleoSpin® 96 Soil DNA Isolation Kit optimized for epMotion® (Macherey-Nagel, Düren, DE) using the epMotion® robotic platform model (Eppendorf) under the manufacturer’s protocol. DNA libraries for Illumina sequencing were prepared with the Kapa HyperPrep kit (KAPA Biosystems, Wilmington, MA, USA). Paired-end 150 bp sequencing was performed using the Illumina NovaSeq apparatus by Novogene (Europe). Low-quality sequences and reads shorter than 100 base pairs were filtered out^43^, and human contamination was filtered out using the BBMap feature of BBTools, with default values. The samples were then processed using the mOTU profiler version 2.5.1 (mOTUs2). This tool works by mapping reads to 10 marker genes (MGs) and then putting them into MG-based operational taxonomic units (mOTUs). The mOTUs were pre-annotated with taxonomic ranks, and the phylogenetic tree was pre-computed^44^.

#### Functional profiling

In addition to gut community characterization, the functional capacity of the metagenomes was characterized by HUMAnN 2.0^45^ revealing pathway coverage and abundances for a total of 532 biochemical modules from the MetaCyc Metabolic Pathway Database^46^. We opted a priori to focus on the abundance of the pathways *PWY6318 L-phenylalanine degradation IV*,* PWY6628 superpathway of L-phenylalanine biosynthesis*,* PWY6629 superpathway of L-tryptophan biosynthesis*,* PWY6630 superpathway of L-tyrosine biosynthesis* and *L-tryptophan biosynthesis* to pursue the relevance of the known neurotransmitters, phenylalanine, tryptophan, and tyrosine^3^. Phenylalanine, tryptophan, and tyrosine were chosen specifically because they have all been implicated in ADHD and also are involved in the gut microbiome^47–50^.

### Covariates

When applicable, BMI was adjusted for, and results were stratified by sex. ADHD has been linked to higher rates of obesity; however, it is unknown whether ADHD contributes to obesity or the other way around. This may be related to dietary habits of people with ADHD, which in turn impacts the gut microbiome as well^51,52^. Furthermore, gut dysbiosis is also associated with higher BMI and is thought to play a role in obesity^53,54^. Lastly, higher BMI is associated with higher inflammation levels^55^.

Moreover, studies have found sex differences in gut microbiome composition^20,54^. There is also expected differences in inflammation levels between males and females because females on average have higher percentage body fat which is associated with increased inflammation^22^. Furthermore, ADHD tends to be overrepresented in males due to bias, however, research suggests that females have been far underdiagnosed^56^.

Also associated with elevated inflammation levels is asthma^12^, which our participants had a higher risk for due to their mothers having doctor-diagnosed asthma^57^. Because of this, asthma was included as a covariate in the nutritional stress test analysis (Supplementary Fig. S1).

### Statistical analysis

**Fig. 1:**
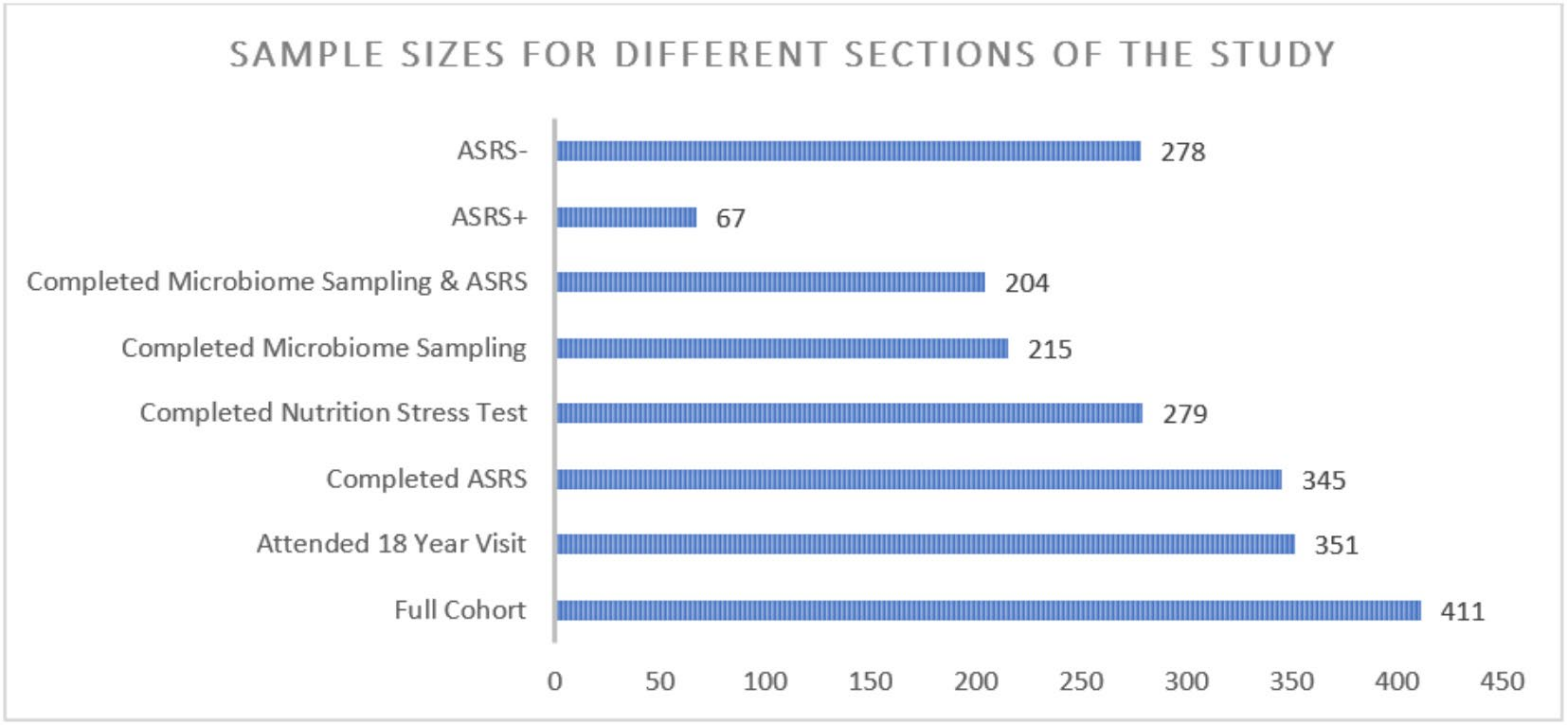
Bar graph depicting the number of participants in each portion of the study.

**Fig. 2:**
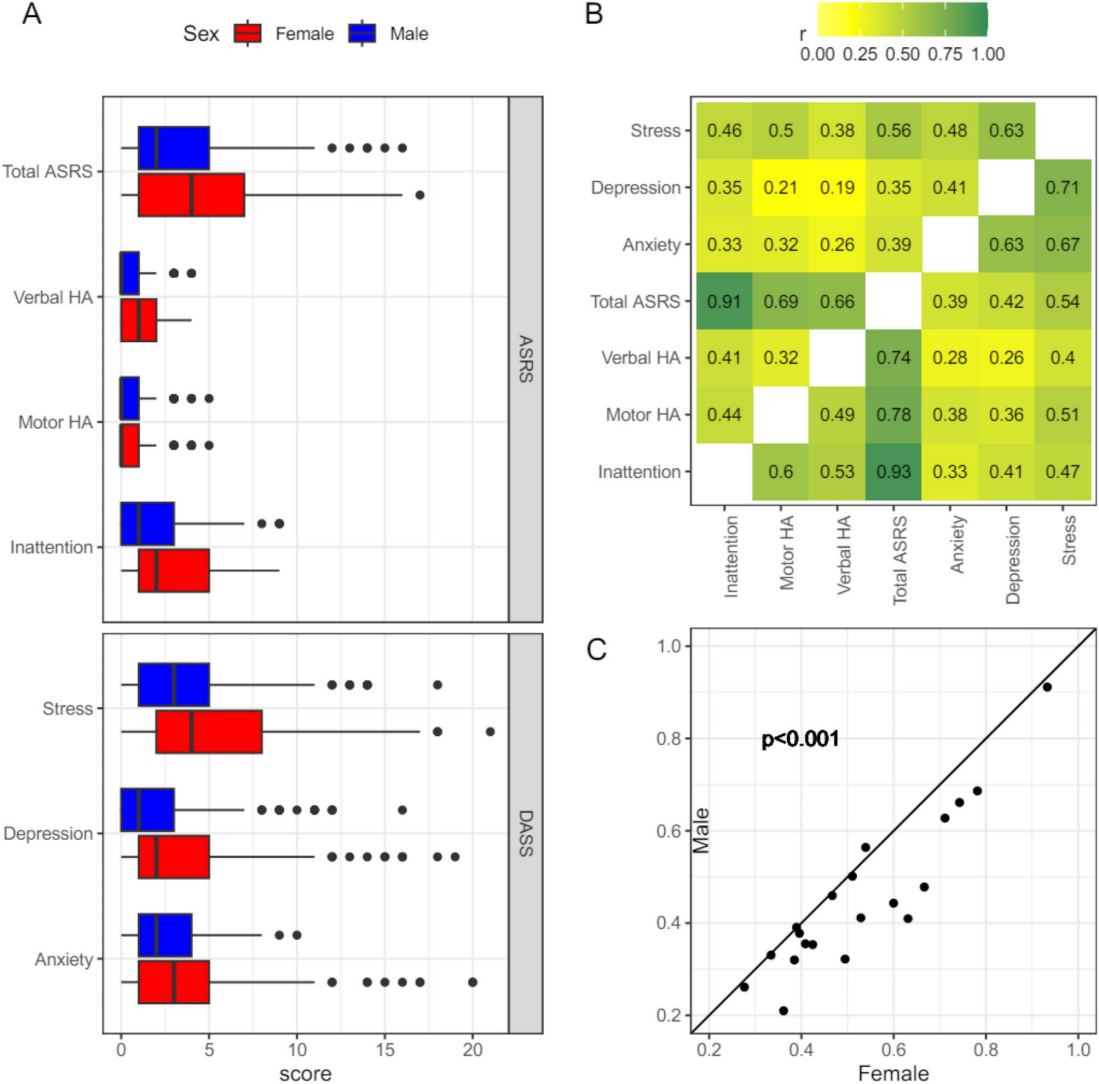
A: Sex-based distribution of scores on ASRS total scale and subscales using continuous measurements B: Pair-wise correlation (Pearson) between continuous scales stratified on sex (Male: above diagonal, Female: below diagonal). C: Correlation coefficients comparing the 21 pair-wise correlations between Sexes (shown in 2B). Linear model fitted to assess the influence of sex on correlation scales. Adult ADHD Self-Report Scale (ASRS), Verbal Hyperactivity/Impulsivity (Verbal HA), Motor Hyperactivity/Impulsivity (Motor HA).

**Fig. 3:**
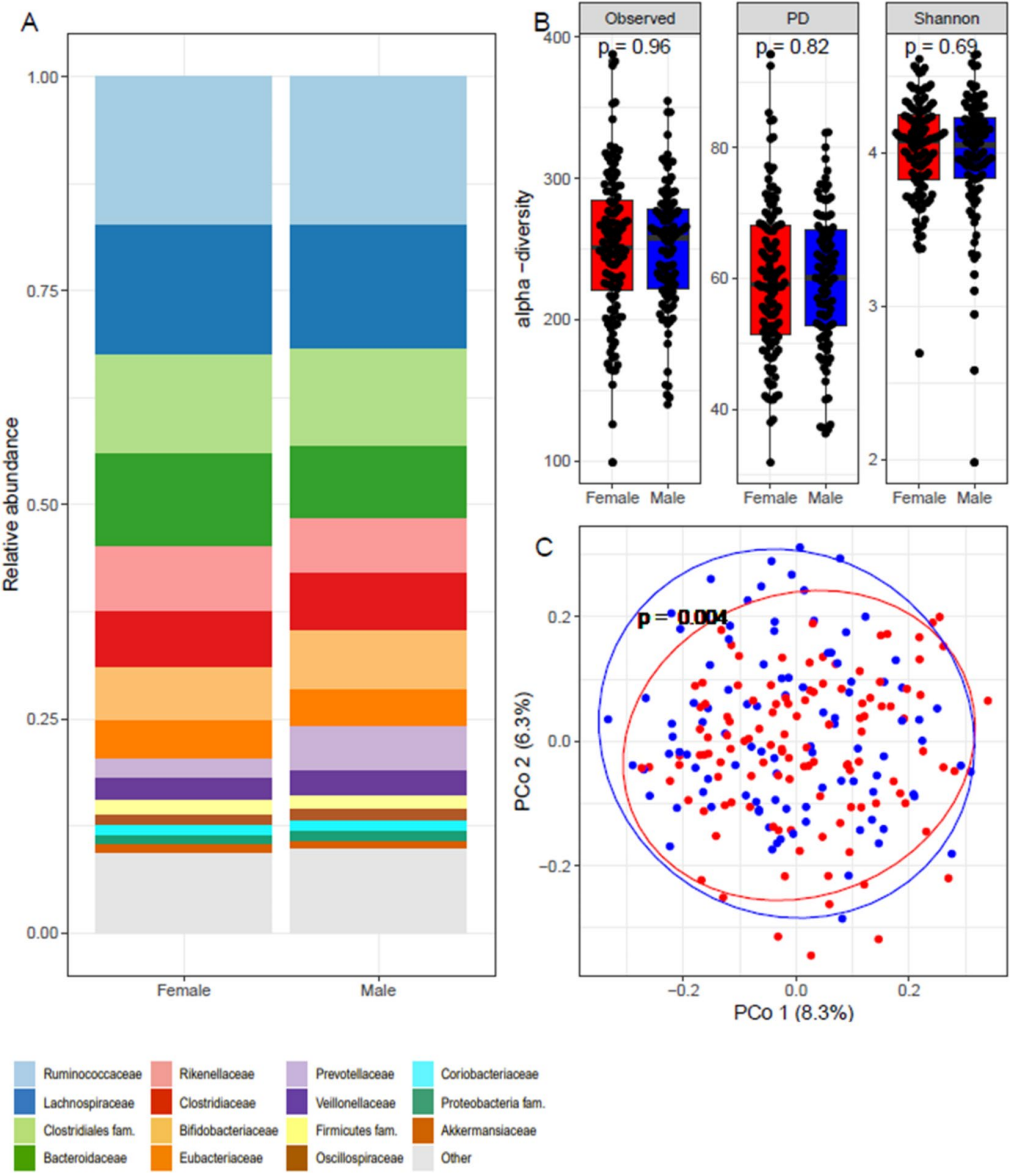
A: Relative abundance of the 15 dominating families. B: Alpha diversity measures for the number of different bacteria (Observed), Faith phylogenetic diversity (PD), and Shannon index (Shannon) for females (red) and males (blue). P-values correspond to Wilcoxon tests. C: Bray-Curtis beta diversity colored according to Sex: females (red) and males (blue). The P-value is for comparing males and females using Adonis.

Not all participants had complete demographic data; because of this, the sample size was smaller for the covariate analyses. We found sex differences in ADHD, co-occurring symptoms, GlycA responses, and gut microbiome diversity in the descriptive analysis (Figs. 1, 2A–C, and 3A–C), and therefore stratified by sex for the subsequent sections. We used continuous analysis for ASRS symptoms at certain points (Figs. 2A–C, 5, and 6C, D) for a couple of reasons. Firstly, we do not have diagnoses and do not intend to look at ADHD from a clinical standpoint but rather explore ADHD symptomology in the general population. Secondly, using continuous measure gave us more statistical power for the analysis. GlycA responses were calculated as an area under the curve (AUC) and an incremental area under the curve (iAUC). AUC is used to estimate the overall GlycA response from baseline to two hours, while iAUC omits the baseline values to account for baseline variation. The two-hour cut-off was chosen because average GlycA levels peaked around 60 min for males and 90 min for females, therefore we analyzed the activation from the nutritional stress test and the initiation of the decline. All statistical analysis was performed in R version 4.2.1.^57^, and the following packages were utilized: *tableone*,* phyloseq*,* corrr*,* caret*,* rabuplot*,* vegan*^58–63^.

Nominal p-values are reported and considered significant under false discovery rate (FDR) correction with q-value less than 0.05.

**Fig. 4:**
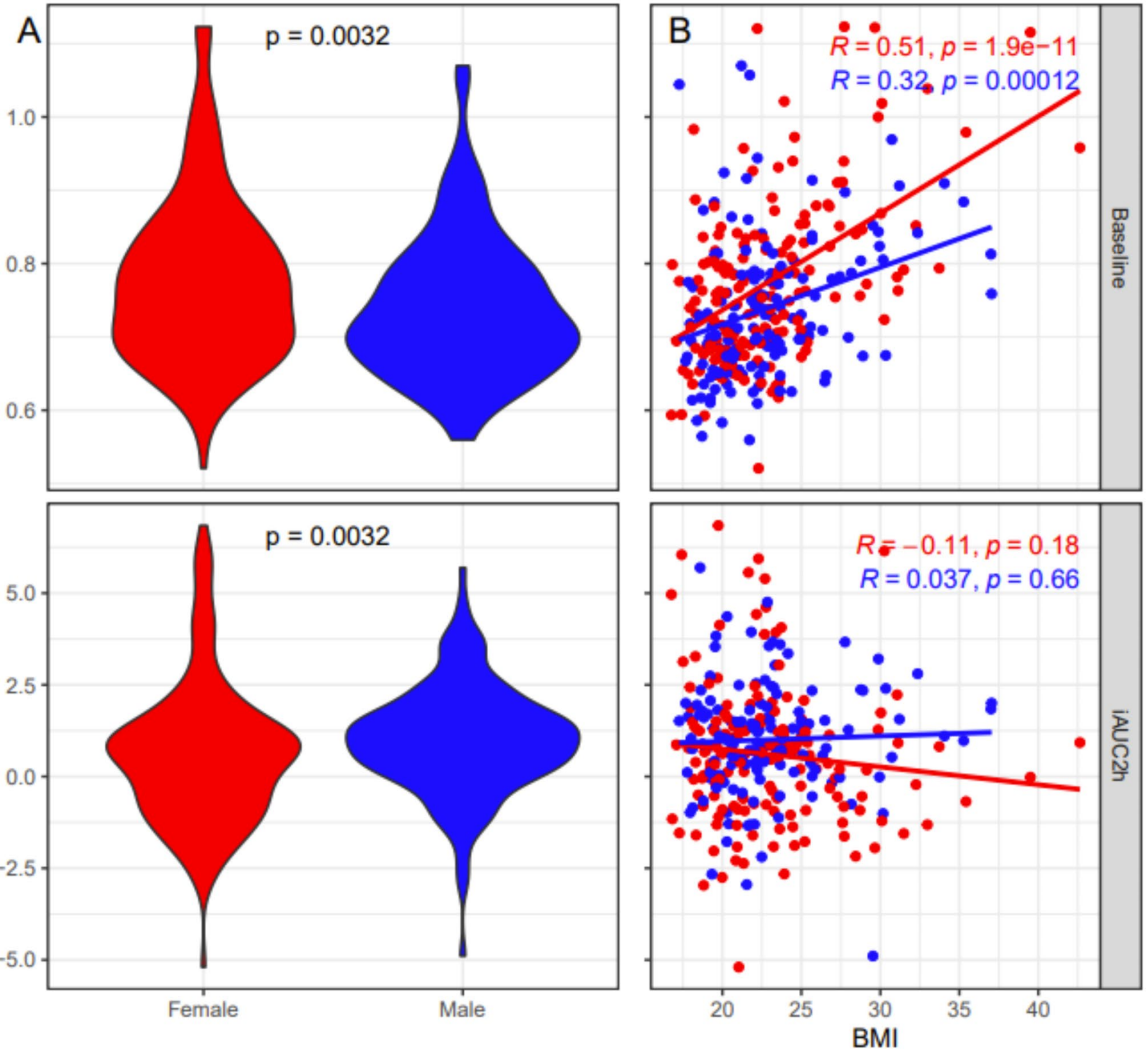
Characterization of glycoprotein A (GlycA) at fasting (baseline) and incremental post-prandial activation after 2 h (iAUC2h). A: Differences between Sexes: females (red) and males (blue). B: Correlation with BMI for each sex.

**Fig. 5:**
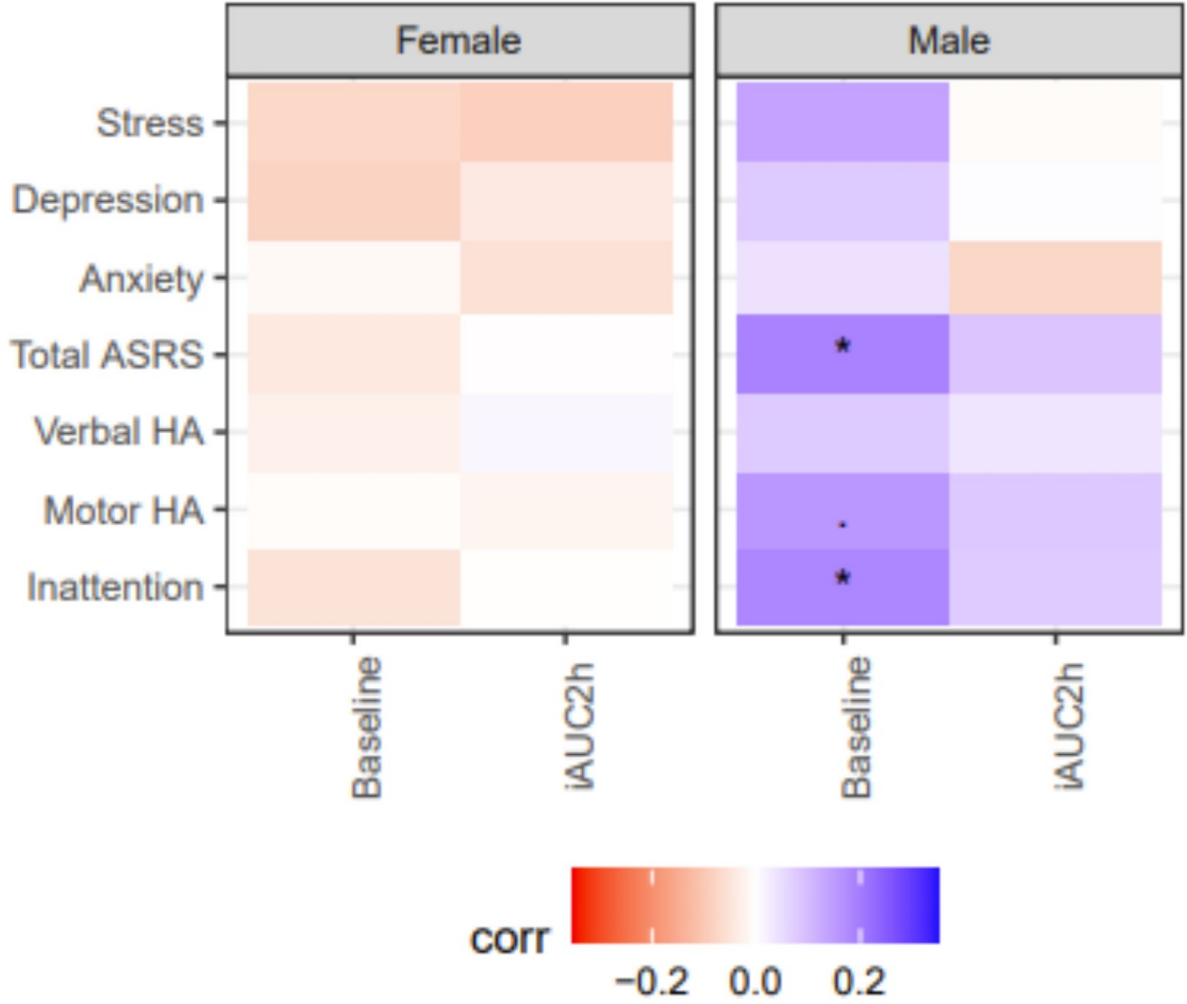
Correlation of continuous scales from ASRS and DASS-21 and baseline (fasting) GlycA and iAUC2h stratified by sex. BMI adjustments were made. Abbreviations: Adult ADHD Self-Report Scale (ASRS), Verbal Hyperactivity/Impulsivity (Verbal HA), Motor Hyperactivity/Impulsivity (Motor HA), incremental area under the curve after 2 hours (iAUC2h). *p* < 0.10, *p < 0.05, **p < 0.01.

**Fig. 6:**
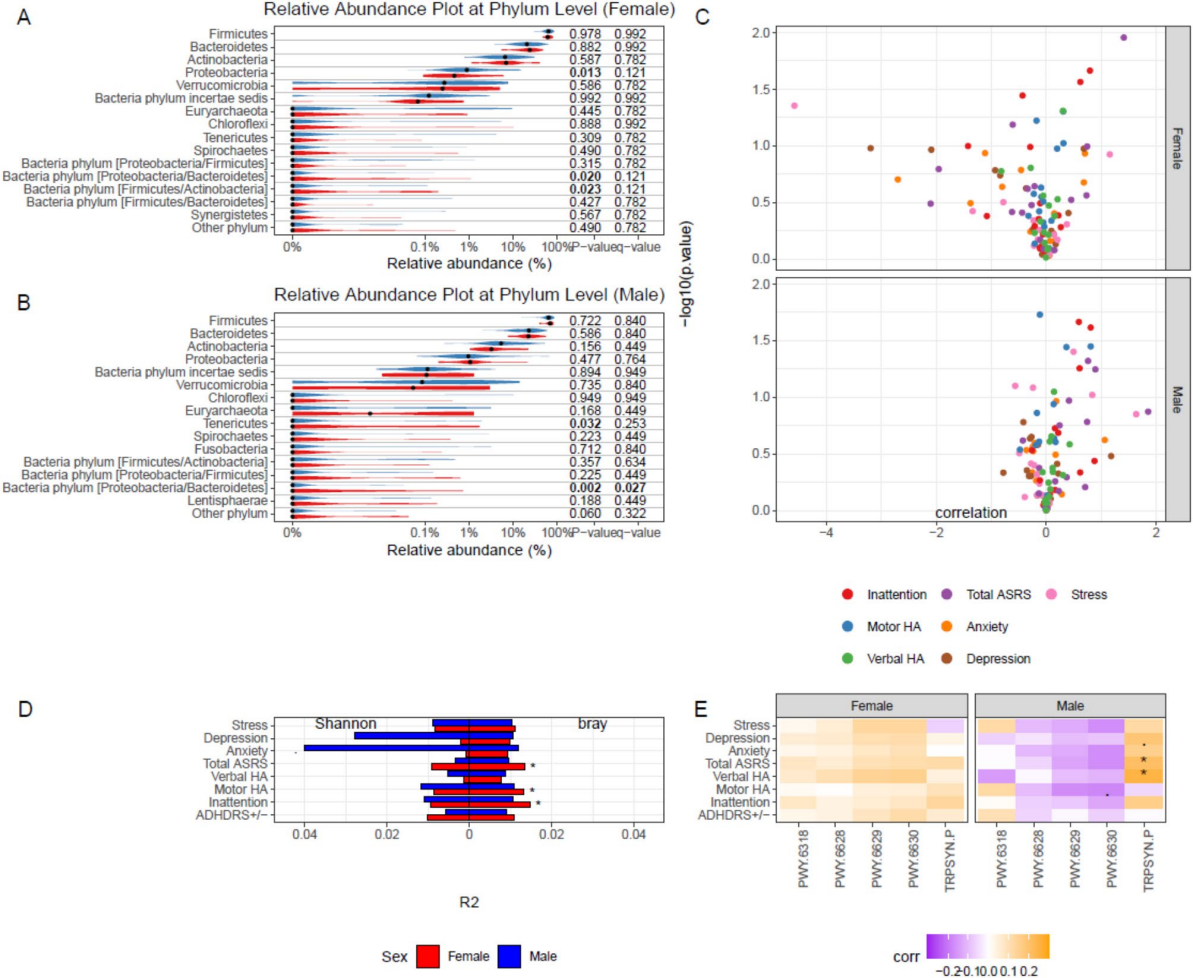
Sex-stratified comparisons of the microbiome with categorical ASRS groups and continuous scales from ASRS and DASS (adjusted for BMI). A: Relative abundance comparison of the dominating 15 phyla in ASRS+ (red) and ASRS- (blue) groups in both sexes. B: Volcano plots of correlation analysis between ASRS and DASS scales and relative abundance at the phylum level in both sexes. C: Alpha diversity (Shannon) and beta diversity (Bray-Curtis) in relation to ASRS and DASS scales, represented in terms of R^2^ values and stratified by sex. D: Abundance of MetaCyc metabolic pathways related to tyrosine, phenylalanine, and tryptophan metabolism (precursors of serotonin) and their correlations with ASRS and DASS scales, stratified by sex. Adult ADHD Self-Report Scale (ASRS), Verbal Hyperactivity/Impulsivity (Verbal HA), Motor Hyperactivity/Impulsivity (Motor HA). *p* < 0.10, *p < 0.05, **p < 0.01.

### Research ethics

The project was approved by the Danish Data Protection Agency (#2015-41-3696). This study was conducted in accordance with the Declaration of Helsinki.

## Results

### Demographic characteristics

Due to limited compliance for different parts of the data collection, the sample size fluctuates. A total of 345 (83.9%) of the 411 participants in the cohort completed the ASRS questionnaire. Measurements of the fasting GlycA were collected from 299 (72.7%) participants, however only 279 (67.9%) participants completed the full four hours of the nutritional stress test. The microbiome data included 215 (52.3%) samples from participants, however, only 204 (49.6%) completed both the microbiome fecal sample collection and the ASRS. For the categorical analysis, there were 67 participants in the psychometrically defined ASRS + group and 278 in the ASRS- group. A comparison of these two groups is shown in Supplementary Table S1.

Baseline characteristics and demographics of adolescents stratified by sex is portrayed in Table 1, showing that there were significant differences in social circumstances and cigarette smoking between the two groups and a nearly significant difference in antibiotic usage in the first 3 years of life.

**Table 1:** Descriptive characteristics and probabilities (p) after T-Test (continuous) or Chi-square (categorical) of study participants stratified by sex.

Demographics	Female	Male	*p*-value
n	178	173	
Gestational age in days: (mean (SD))	281.96 (9.80)	280.08 (10.09)	0.078
Delivery mode			0.036
Acute C-section	18 (10.1)	28 (16.2)	
Natural birth	141 (79.2)	137 (79.2)	
Planned C-section	19 (10.7)	8 ( 4.6)	
Mother’s age at 2 years: (mean (SD))	32.49 (4.67)	31.73 (4.39)	0.126
Used antibiotics at 3 tears: Y(%)	153 (86.0)	155 (89.6)	0.380
Social circumstances (mean (SD))	0.12 (1.05)	– 0.06 (0.96)	0.103
Living with parents: Y(%)	165 (92.7)	163 (94.2)	0.718
Urban household: Y(%)	72 (43.6)	69 (42.9)	0.976
Has a Cat or Dog: Y(%)	130 (73.0)	126 (72.8)	1.000
BMI: (mean (SD))	23.12 (4.26)	22.87 (3.93)	0.570
Transport time: (mean (SD))	2.35 (2.07)	2.07 (1.61)	0.294
Ever smoked: Y(%)	89 (50.0)	91 (52.6)	0.703
Asthma at 18 years	44 (24.7)	44 (25.4)	0.975

*Social circumstances are calculated using the mother’s education at birth, the father’s education at birth, and household income at birth^64^.

On average, females had higher total ADHD scores, verbal hyperactivity/impulsivity, motor hyperactivity/impulsivity, and inattention scores on the ASRS apart from motor hyperactivity/impulsivity scores. Similar findings were shown for depression, anxiety, and stress scores on the DASS, with females reporting higher scores on all three categories (Fig. 2A). Correlation matrices of ADHD symptom presentations, depression, anxiety, and stress symptoms within participants, stratified by males and females, showed that overall, females have a stronger correlation within their group between most of the scales when compared to males (Fig. 2B). When directly comparing the pairwise correlation matrices of male participants to female participants, there was a significant difference between the females, suggesting that females faced a higher burden from co-occurring symptoms (Fig. 2C).

There was no major variation in the relative abundance of bacteria at the family level, as well as in alpha diversity between females and males in the cohort (Fig. 3A, B). However, Bray-Curtis analysis of beta diversity revealed that the bacterial community composition within the male and female clusters was significantly different (*p* = 0.004) (Fig. 3C).

### Nutritional stress test

When comparing males and females, there was a significant difference in both fasting and post-prandial levels of systemic inflammation, which is calculated using GlycA measurements (Fig. 4A). BMI was significantly associated with GlycA at fasting in both males and females; marginally stronger for the post-prandial signal, however the net incremental post-prandial inflammation, as reflected by iAUC, did not show any association for either sex (Fig. 4B). When stratified by sex, there was no relationship between any scales of mental health symptoms and levels of inflammation in females. For males, there was a significant positive correlation between fasting AUC GlycA levels and total ASRS scores and inattention. There was also a significant positive correlation between total ASRS scores and inattention in males (Fig. 5). This same analysis was done adjusting for both asthma and BMI, however, there were no significant changes (Supplementary Fig. S1).

### Gut microbiome and metacyc metabolic pathways

Relative abundance analysis was performed at the phylum level comparing abundances of bacteria phyla between the ASRS + and ASRS- groups, which were categorized based on ASRS scores. In females, we found that the relative abundance of two unspecified phyla (shown as Proteobacteria/Bacteroidetes and Firmicutes/Actinobacteria) and Proteobacteria were significantly higher in the ASRS + group, however, these results did not survive multiple testing (Fig. 6A). Proteobacteria/Bacteroidetes phylum was classified as unknown by the reference database because of genetic similarities between both phyla displayed. There were taxa from each phylum that were potential matches at every taxonomic level, resulting in ambiguity. The Firmicutes/Actinobacteria phylum similarly showed similarities to both phyla, however, it was assigned to the Actinobacteria class *Coriobacteriia*, and further classified under the genus *Collinsella*, which typically falls within Actinobacteria, indicating that although the sequence has some features resembling Firmicutes, it is more closely related to Actinobacteria. In males, the relative abundance of the phyla Tenericutes and Proteobacteria/Bacteroidetes was significantly higher in the ASRS + group. Tenericutes does not survive multiple testing, however, Proteobacteria/Bacteroidetes remains statistically significant even after multiple testing (q = 0.027) (Fig. 6B).

Proteobacteria/Bacteroidetes is still significantly higher in the ASRS + group without sex stratification (Supplementary Fig. S2). At the family level, there were no significant bacteria families when looking at relative abundance in males and females independently or the participants overall (Supplementary Figs. S3–S5). Furthermore, at the phylum level, there was no significant association between the gut microbiome and inattention, ASRS scores, or DASS scores in neither males nor females based on a linear model stratified by sex (Fig. 6C). There were weak associations between beta diversity and ASRS scores, motor hyperactivity/impulsivity symptoms, and inattention symptoms however, nothing survived multiple testing correction (Fig. 6D). The high-level analysis on alpha and beta diversity demonstrate that there is no community level differences between ASRS+/−. In the more targeted approach, we find weak indications of differences from the 14th most abundant phylum. On a top 15 family level resolution analysis, we merely investigate families from the dominating phyla and are hence limited in ability to discover differences occurring from the bacteria less abundant in the kingdom.

MetaCyc metabolic pathway abundance of tyrosine, phenylalanine and tryptophan was also analyzed in relation to mental health symptoms separately in males and females. For females, there was no significant relationship between any of the pathways and mental health symptoms, however, for males, the L-tryptophan biosynthesis pathway was significantly correlated with higher total ASRS scores and higher verbal hyperactivity/impulsivity (Fig. 6E).

## Discussion

### Primary findings

This study aimed to elucidate the relationship between the gut microbiome and ADHD symptoms, investigate fasting and postprandial inflammation after a standardized meal challenge using GlycA as a potential mediator, and explore ADHD, anxiety, depression, and stress symptom severity in relation to inflammation and the gut microbiome. Sex-stratified analyses showed that in males, but not in females, the severity of ADHD symptoms and verbal hyperactivity/impulsivity were significantly correlated with the biosynthesis pathway of tryptophan. Furthermore, fasting GlycA and 2 h post-prandial GlycA were positively correlated with inattention and total ADHD symptoms severity in males. This study shows potential sex differences in both ADHD symptomology and an association between postprandial inflammation and ADHD, suggesting that metabolic pathways involved in post-prandial inflammatory responses might play a role in ADHD symptomology in males, and should be a target of future ADHD research.

### Implications and limitations

This is a cross-sectional cohort study, and as such no causal linkage can be determined. Although an association between ADHD symptom severity and GlycA was observed, the directionality is unclear. One limitation of our study is the reliance on an established data set, which restricted our control over the selection of measures. Consequently, we were confined to the measures that were already collected, potentially impacting the comprehensiveness of our findings. For instance, although there are advantages to using GlycA as our marker for inflammation, there are inconsistencies in the obtained values depending on if it was measured using a high volume NMR metabolomics research platform versus the LabCorp “clinical” GlycA assay, however, there are statistical adjustments that can be made to get more accurate results^38^. This data set was also composed of individuals who all had a mother with asthma. As mentioned previously, asthma has a link both to inflammation and ADHD. While this connection may limit the generalizability of our findings, 262 million people suffer from asthma globally^65^, underscoring the importance of considering comorbid conditions in health research. The induction of post-prandial inflammation were under a specific combined meal focusing on saturated fats and sugar as proposed by Stroeve et al.^36^, therefore, other nutritional stress testing setups may give different results.

Furthermore, we examined a cohort with relatively mild symptoms and no clinical diagnosis. The study relies on the validity of the ASRS questionnaire, which has been previously demonstrated^24,25^. As with all self-reporting, there are risks of bias leading to either under- or overestimation of symptoms^66^. This likely applies to the DASS-21 questionnaire as well^67^. However, this also allows us to assess a population more consistent with the general population, as the ASRS score distribution for our cohort was consistent with another Danish screening study utilizing the ASRS^30^. Moreover, for select analyses, we treated ASRS symptoms as a continuous variable. Although ADHD is clinically diagnosed using categorical criteria, and most of the existing literature employs categorical measures, we opted for a continuous approach to enhance statistical power. Although this decision may limit the generalizability and comparability of our findings with existing research, it allows for a more nuanced analysis of real-world symptom distributions.

Although the study included important covariates, analysis on the participants’ diets was not performed. The gut microbiome is heavily influenced by diet^68^, and so it is impossible to determine what is driving the variation observed in the gut microbiomes. According to a 2019 systematic review and meta-analysis of 14 observational studies, people with ADHD tend to have dietary patterns characterized by foods high in saturated fats and refined sugars, whereas diets high in fruits, vegetables, and whole grains are associated with lower rates of ADHD diagnosis and less severe ADHD symptoms^69^. Therefore, in future studies, it would be useful to look at the relationship between certain dietary patterns and the gut microbiome in people with ADHD.

A notable limitation of this study is the discovery power based on the number of included individuals, as well as the low number of cases. While metagenomic data allows for high resolution, we are not able to pursue statistical analysis on this level and consequently agglomerates to a higher taxonomic similarity. However, metagenomics provides biochemical resolution which is a strength.

Furthermore, the small sample size increases the chance of type ll error, potentially leading to overestimating results that would not be found with a larger cohort^70^. It also is harder to generalize results to the general population with such a small sample size, as the sample may not contain the same variability^71^. Lastly, this study is done in Denmark, and therefore the findings may not be applicable to countries with different cultures and contexts.

Despite the small cohort, this study contributes to ADHD research by providing insight into the relationship between the gut microbiome, fasting and postprandial inflammation, ADHD presentations, ADHD symptoms severity, and co-occurring anxiety, depression, and stress symptom severity.

### Fasting and postprandial inflammation

The metabolism of the serotonin precursor tryptophan is associated with another potential driver of ADHD symptomology, systemic inflammation^72^. Inflammatory cytokines are involved in serotonin and dopamine pathways^73^, two major neurotransmitters in mood regulation and mental disorders^74^. Elevated inflammatory cytokine levels in ADHD versus healthy controls have been found in previous literature^75^. In the current study, fasting and post-prandial GlycA levels were significantly correlated with higher ASRS scores, motor hyperactivity/impulsivity, and inattention in males, but not in females, whereas GlycA was positively correlated with BMI in both males and females. It is important to acknowledge the bias in our sample, as all participants have mothers with asthma, a condition strongly linked to inflammation. Asthma has a genetic component and a high comorbidity rate with ADHD and other inflammatory disorders^12^. Consequently, the generalizability of our findings is limited.

Studies have identified that maternal inflammation is associated with ADHD and other neurodevelopmental disorders in childhood, suggesting that the development of ADHD, has already been set in motion during gestation^41,42,76,77^. There are several mechanisms by which inflammatory cytokines from the mother can affect the developing brain, some through direct interaction with the fetal brain and others more indirectly through triggering epigenetic changes to the placenta or initiating a fetal inflammatory response^41^. Rosenberg et al.^42^ found a dose-dependent relationship between hs-CRP levels during pregnancy and offspring’s risk of ADHD. Considering maternal inflammation as a potential etiological factor for ADHD, the effects of inflammation on ADHD development likely occur long before the developmental timeframe that this study explores at age 18 years. While numerous animal studies have established a clear link between maternal inflammation and elevated inflammation levels in offspring persisting into adulthood^78–80^, longitudinal studies examining this phenomenon in humans remain limited. Notably, a 2016 study revealed that prenatal maternal depression—an inflammation-related condition—correlates with increased inflammation levels in offspring at the age of 25, independent of whether the offspring experienced depression in adulthood. This finding underscores the notion that prenatal developmental factors can exert significant and lasting effects on the health of offspring^81^. Although inflammation levels in adulthood do not affect the development of ADHD, it could potentially affect symptom severity^11^, and contribute to the multimorbidity burden that is common in people with ADHD^82^. Despite finding no major associations between ADHD and inflammation in our study, the inflammatory response may be contributing to ADHD symptomology at other stages of development that we did not explore. Furthermore, the associations that we did find could be attributed to lifestyle habits commonly portrayed in individuals with ADHD, which may also have an influence on inflammation levels^40,51,83^, however, the directionality of this interaction is still uncertain.

### Co-occurring anxiety, depression, and stress symptoms

The gut microbiome is known to influence serotonin and dopamine production as well as potentially contribute to chronic inflammation^47,74,84^. Furthermore, as the gut-brain-axis is a bidirectional pathway, the brain can also affect the gut, with multiple neurotransmitters being implicated in gastrointestinal disorders^74^. While the main focus of this study was to explore the connection between ADHD, the gut microbiome, and fasting and postprandial inflammation, we also explored symptoms of anxiety, depression, and stress, since research suggests that they could potentially share the same mechanisms revolving around inflammation and dopamine^13,74^.

In general, we observed strong associations between anxiety, depression, and stress symptoms with ADHD symptoms. Similar trends of associations between co-occurring symptoms were seen with the microbiome and inflammation, however, the signals were weaker for anxiety, stress, and depression in comparison with symptoms of ADHD. It should be noted, however, that we did not have diagnoses for depression and anxiety, therefore, these findings should not be viewed as comorbidities but rather overlap of symptomology.

Sub-clinical mental health symptoms are still influential to health, well-being, and society. A 2021 Dutch study found not only that subclinical depressive symptoms were highly prevalent among adolescents, but also that it costs over €42 million annually. Sub-clinical depression is linked to obesity, physical illness, and suicide, along with increasing the risk for developing clinical depression later in life^85^. Stress is also associated with these disorders. Further, it has been shown that having ADHD as a child contributes to childhood stress, and having experienced stressful events as a child potentially increases the occurrence of ADHD and symptom severity. Stress is also correlated with inflammation and immune dysregulation^13^. Serotonin and dopamine have been implicated in many neuropsychiatric disorders and are also implicated in depression, anxiety, and ADHD^74,86^.

### COPSAC_2000_ birth cohort

The participants in the COPSAC_2000_ Birth Cohort all have a mother who has asthma and are therefore not totally representative of the Danish population. This could be why there is a higher-than-average amount of participants in the ADHD group in the cohort, with about 20% meeting the criteria for ADHD based on the ASRS, whereas the global average is closer to 6%^1^. It could also be because although the ASRS has been found to be quite consistent with ADHD diagnoses, it is not an official method of diagnosing ADHD^29^, and a health professional may not have included all these cases.

Interestingly, there were more females than males in the cohort that were placed in the ASRS + group. This is contradictory to what is commonly found in the literature. Previous findings support a 1:4 ratio of males to females in childhood ADHD cases. However, in adults, it is much closer to 1:1. It is known that bias in diagnosing has resulted in females being overlooked by mental health professionals due to less obvious symptom presentations^87^. This bias is avoided by using the ASRS. In the ASRS validation study by Hoeffding et al., while males were more represented in the ADHD group as a whole, males had more hyperactive symptoms while females had more impulsivity/hyperactivity symptoms. However, their study has a significant sampling bias since all participants were blood donors, reducing its generalizability. Further, the median age of participants was 40 years old^30^, and it is not unexpected for there to be generational shifts in self-report symptoms or diagnoses^88^. A study performed in the Polish setting on ADHD in adolescents using the ASRS found that ADHD was not only much higher in prevalence than previously reported, but there were also no significant sex differences, although females screened positively slightly more than males did^89^.

Furthermore, the increased awareness of ADHD on social media, specifically targeting females, may contribute to an overreporting of symptoms. Viewing ADHD related content on social media has been demonstrated to increases the likelihood of an individual with no prior diagnosis of ADHD attributing general symptoms to ADHD diagnosis^90^. ADHD diagnoses are becoming more prevalent in general, in large part as a result of the increased awareness of the condition opening the door for more minority groups and women who have traditionally been underrepresented to seek out treatment^91^. Taking all of this into account, it is plausible that females would score higher on the ASRS, especially in the adolescent age range, however, it remains uncertain whether these participants would qualify as clinical diagnoses.

## Conclusion

This study expands the current knowledge on the gut-brain axis in ADHD as well as symptom severity of ADHD, anxiety, stress, and depression by investigating specific microbial differences in the gut as well as fasting and postprandial systemic inflammation measured as GlycA levels in young adults. The study did not find significant gut microbiome associations with ADHD presentations, ADHD symptoms, or co-occurring symptoms. However, there was an association between the GlycA response during a nutritional stress test and ADHD symptoms, specifically in males. Furthermore, in males, but not in females, the severity of total ADHD symptoms and verbal hyperactivity/impulsivity were significantly correlated with the biosynthesis pathway of tryptophan. This study is on a moderate-sized Danish cohort; therefore, these findings need to be replicated in larger, prospective cohorts in different contexts for them to be conclusive.

## Electronic supplementary material

Below is the link to the electronic supplementary material.

Supplementary Material 1

## Data Availability

The COPSAC2000 metagenomics data can be found in the Sequence Read Archive (SRA) under the accession number PRJNA916259. The metagenome-assembled genomes generated can be found at DDBJ/ENA/GenBank under the accession number PRJNA1026956. According to the Danish Data Protection Act and European Regulation 2016/679 of the European Parliament and the Council (GDPR), data involving the personal privacy of project participants cannot be publicly available. Research collaborations are open, and data can be accessed via joint research collaborations by contacting the COPSAC Data Protection Officer, Dr. Ulrik Ralfkiaer, at administration@dbac.dk.
