# Supplementary material for: Associations of the gut microbiome and inflammatory markers with mental health symptoms: a cross-sectional study on Danish adolescents
Source: Sci Rep. 2025 Mar 26;15:10378. doi: 10.1038/s41598-025-94687-7 (PMC11947166; doi:10.1038/s41598-025-94687-7)

Supplementary Materials

Supplementary Table S1: Descriptive Characteristics and Probabilities (p) After T-Test (continuous) or Chi-square (categorical) of Study Participants Stratified by ASRS+/-

Demographics	ASRS+	ASRS-	p-value
n	67	278	
Male: N (%)	25 (37.3)	145 (52.2)	0.041
Gestational Age in Days: mean (SD)	281.10 (9.35)	280.91 (10.08)	0.888
Delivery Mode			0.337
Acute C-Section: N (%)	5 (7.5)	39 (14.0)	
Natural Birth: N (%)	57 (85.1)	217 (78.1)	
Planned C-Section: N (%)	5 (7.5)	22 (7.9)	
Mother's Age at 2 Years in Years: mean (SD)	32.94 (4.65)	31.92 (4.55)	0.112
Antibiotics in First 3 Years: N(%)	58 (86.6)	245 (88.1)	0.886
Social Circumstances*: mean (SD)	0.02 (0.94)	0.03 (1.03)	0.974
Living with Parents: N(%)	62 (92.5)	260 (93.5)	0.985
Urban Household: N(%)	33 (51.6)	107 (41.6)	0.196
Has a Cat or Dog: N(%)	45 (67.2)	207 (74.5)	0.292
BMI: mean (SD)	23.97 (4.43)	22.79 (4.01)	0.035
Asthma at 18 Years	19 (28.4)	69 (24.8)	0.66
Fecal Sample Transport Time in Days: mean (SD)	2.06 (1.47)	2.25 (1.95)	0.573
Smoked at Least 1 Cigarette: N(%)	44 (65.7)	133 (47.8)	0.013
Completed DASS-21	67 (100.0)	277 (99.6)	1.000
Completed Microbiome Analysis	35 (52.2)	169 (60.8)	0.254
Completed Nutrition Stress Test	55 (82.1)	228 (82.0)	1.000

Supplementary Figure S1: Correlation of continuous scales from ASRS and DASS-21 and baseline (fasting) GlycA and iAUC2h stratified by sex with BMI and asthma adjustments.

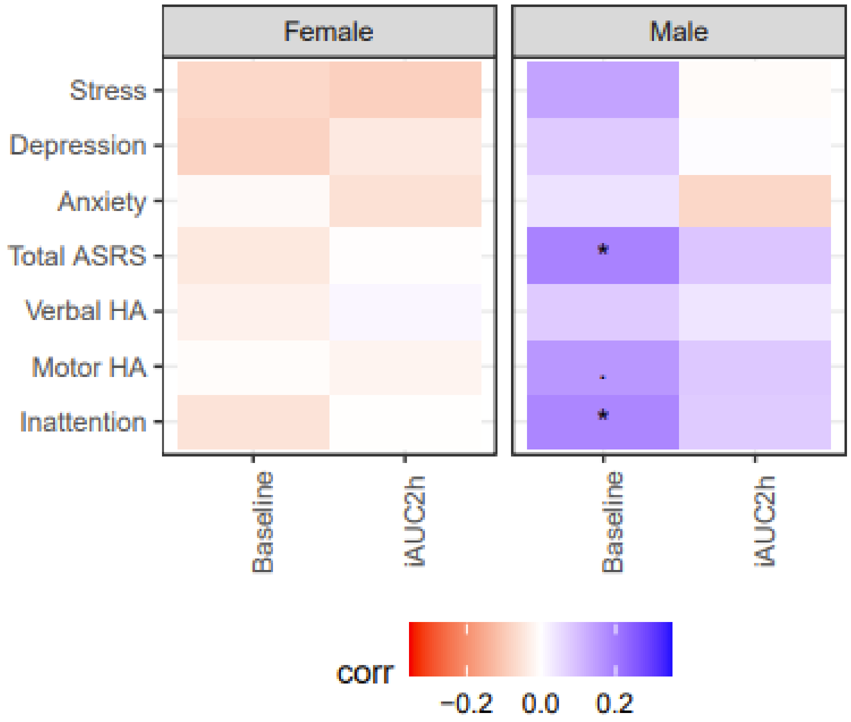

Abbreviations: Adult ADHD Self-Report Scale (ASRS), Verbal Hyperactivity/Impulsivity (Verbal HA), Motor Hyperactivity/Impulsivity (Motor HA), incremental area under the curve after 2 hours (iAUC2h).

.p<0.10, *p<0.05, **p<0.01

Supplementary Figure S2: Comparisons of the microbiome with categorical ASRS groups and continuous scales from ASRS and DASS with BMI adjustment.

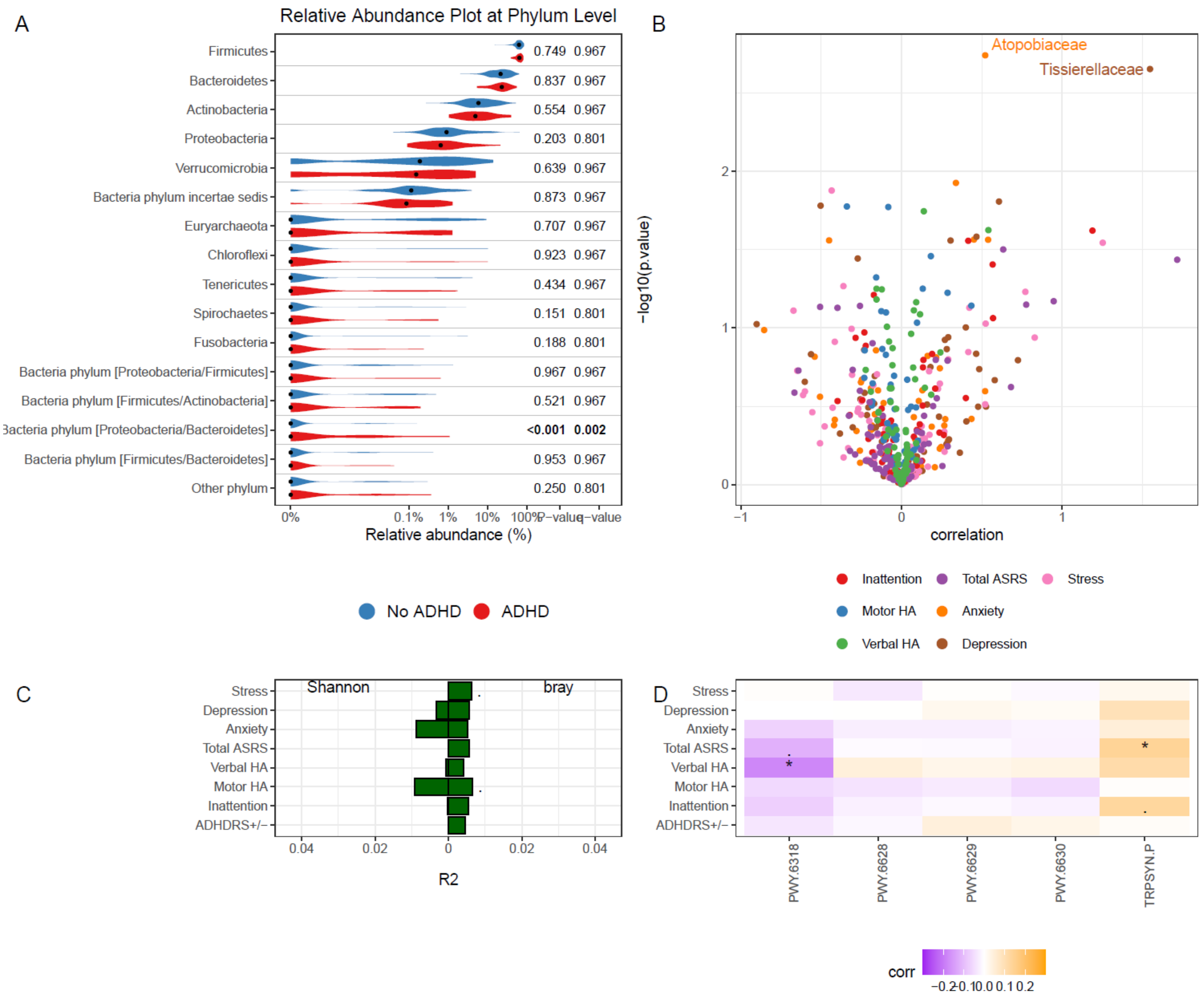

A: Relative abundance comparison of the dominating 15 phyla in ASRS+ (red) and ASRS- (blue) groups. B: Volcano plots of correlation analysis between continuous ASRS and DASS scales and relative abundance at the phylum level. C: Alpha diversity (Shannon) and beta diversity (Bray-Curtis) in relation to continuous ASRS, categorical ASRS groups, and DASS scales, represented in terms of R2 values. D: KEGG pathway abundance of tyrosine, phenylalanine, and tryptophan relevant pathways (precursors of serotonin) correlations with continuous ASRS scales, categorical ASRS groups, and DASS scales. Abbreviations: Adult AHD Self-Report Scale (ASRS), Verbal Hyperactivity/Impulsivity (Verbal HA), Motor Hyperactivity/Impulsivity (Motor HA). .p<0.10, *p<0.05, **p<0.01

Supplementary Figure S3: Relative abundance comparison of the dominating 15 families in ASRS+ (red) and ASRS- (blue) groups in females.

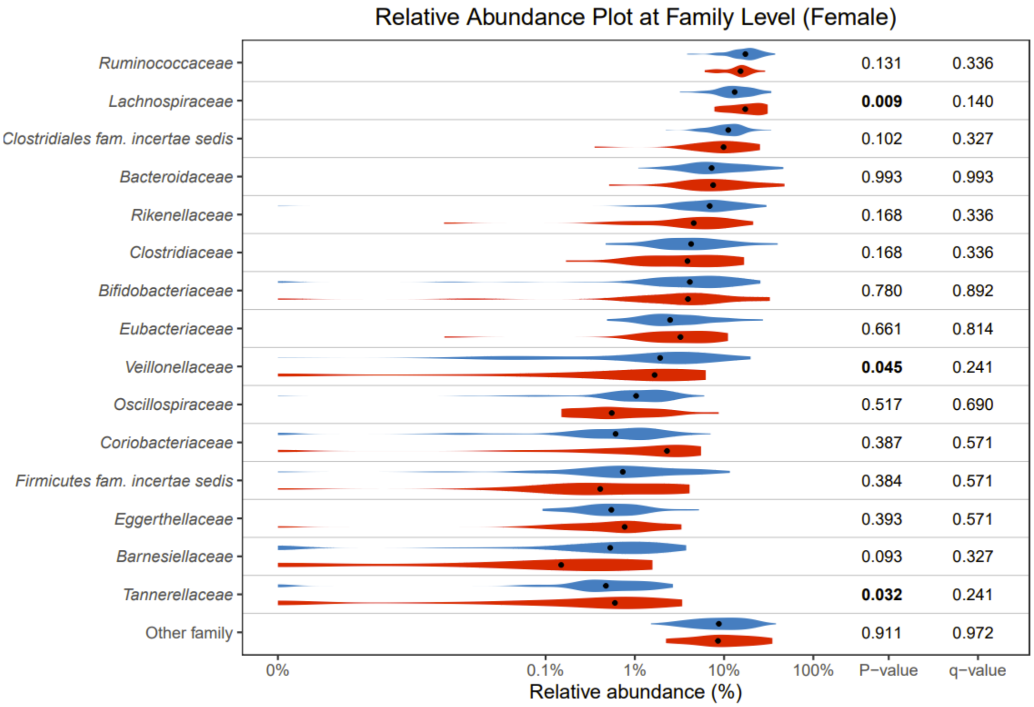

Supplementary Figure S4: Relative abundance comparison of the dominating 15 families in ASRS+ (red) and ASRS- (blue) groups in males.

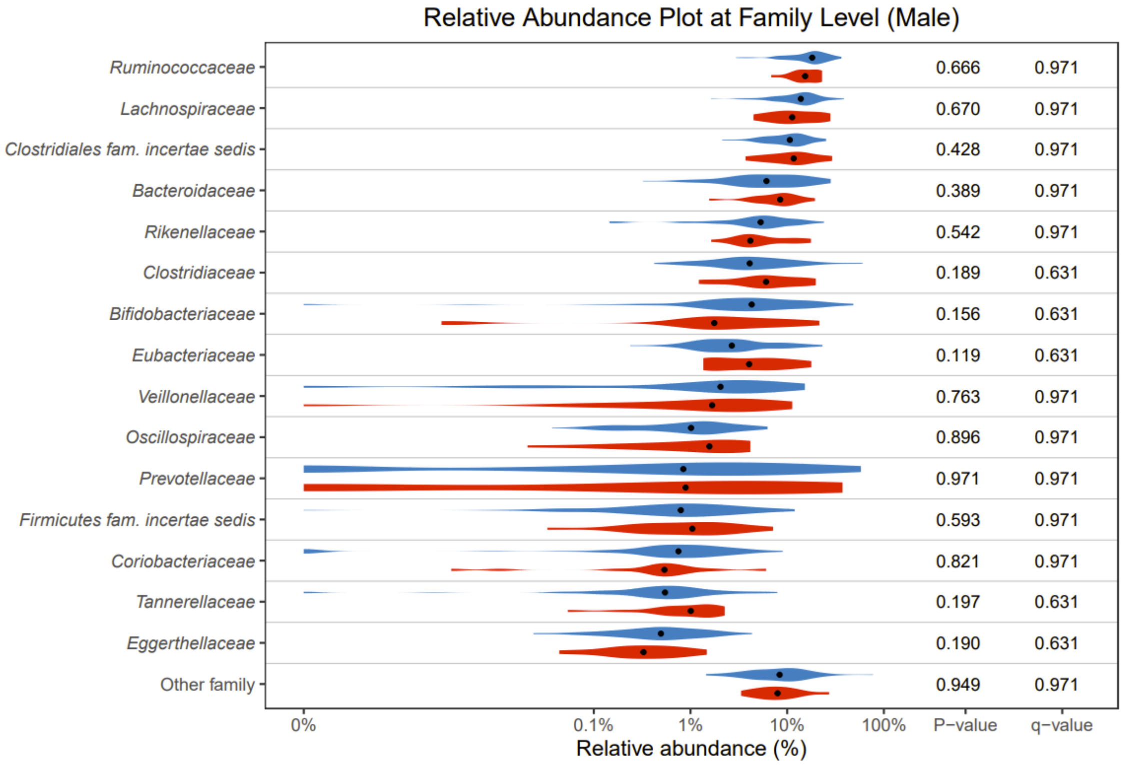

Supplementary Figure S5: Relative abundance comparison of the dominating 15 families in ASRS+ (red) and ASRS- (blue) groups overall.

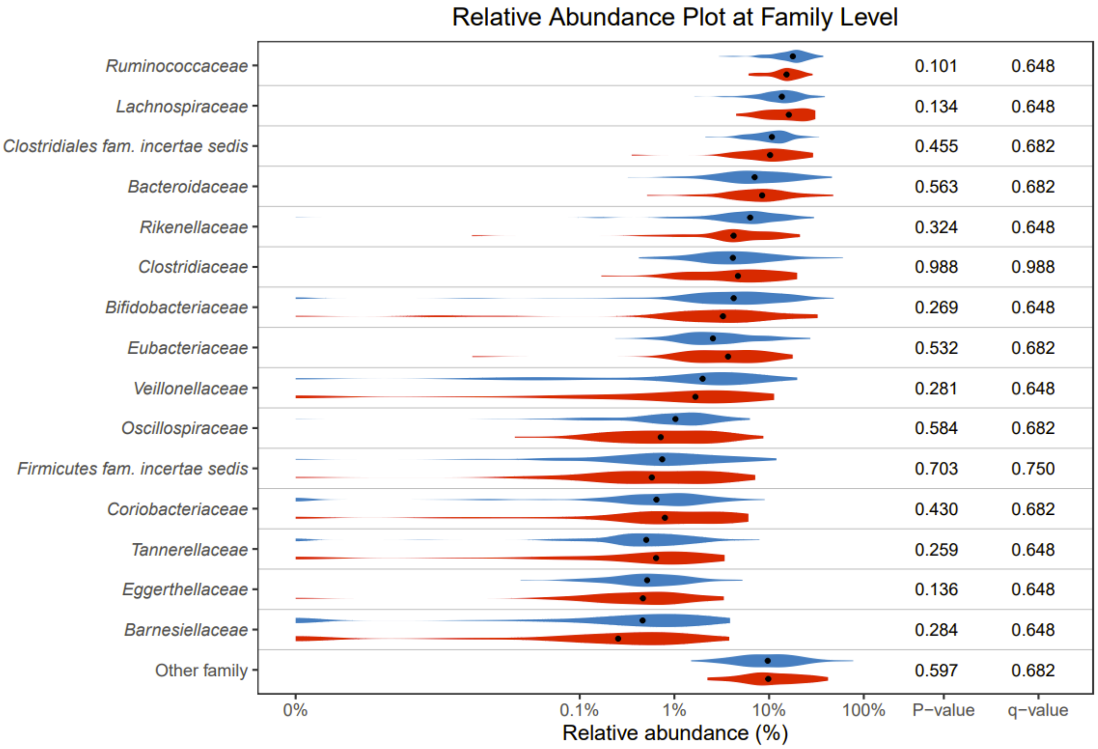

Supplement: Supplementary file 1 — Supplementary Material 1 [file 41598_2025_94687_MOESM1_ESM.pdf]
